# The silent crisis: unveiling the global health burden of psychotherapy delays through the Mental Health Access Burden Index (MHABI)

**DOI:** 10.1192/j.eurpsy.2026.10292

**Published:** 2026-07-31

**Authors:** K. Noor

**Affiliations:** Foundation of Clinical Research, Harvard Medical School, Boston, United States

## Abstract

**Introduction:**

The global landscape of mental health is defined by a profound paradox: while clinical understanding and therapeutic modalities have reached unprecedented levels of sophistication, the actual ability for a person to access care remains obstructed. This crisis has evolved into a rapidly growing epidemic, with mental health conditions now soaring past one billion people worldwide (WHO, 2025).

As a result, mental health is now a leading cause of disability globally (Benefit Representatives of America, 2023). Systemic failures in follow-up care contribute to mortality risk, as worsening symptoms during extensive wait periods increase readmission rates (Pederson et al., 2016). By quantifying these invisible obstacles, the Mental Health Access Burden Index (MHABI) moves focus from clinical symptoms to structural barriers, humanizing data to ensure no patient is “lost in transition.”

**Objective:**

The objective outlines the development and implementation of the Mental Health Access Burden Index (MHABI), a composite measurement framework created to quantify the multidimensional burden resulting from delays in accessing mental health care across institutional, individual, and organizational levels.

**Methodology:**

The MHABI (0–100) is an algorithmic framework synthesizing Clinical Burden (Wait Time/DALYs), Economic Impact (Missed Work/School), and Crisis Risk (ER/Suicide history). Results are categorized via a Burden Score Reference Scale: Low (0-39), Moderate (40-69), High Critical (70-85), and Critical (86-100). The provisionally patented methodology introduces a paradigm shift from linear sorting (the “Deli Counter” model) to Burden-Based Stratification across four risk tiers. It employs Diagnostic Sub-Scores to identify why a score is high and Risk Amplification Logic, a conditional multiplier that elevates the composite score when high-risk indicators co-occur, reflecting real-world clinical complexity.

**Results:**

Simulated scenarios show MHABI identifies patients “waiting in line” who require immediate intervention. It addresses the “Deli Counter” blind spot where Patient A (low acuity) and Patient B (high-risk/unemployed) are treated identically due to a “False Equivalence” in wait-time metrics. Prompt, score-prioritized access reduces symptom severity and duration by up to 87%. This targeted approach preserves socioeconomic stability, functional independence, and alleviates clinician cognitive load.

**Conclusions:**

The MHABI bridges the gap between the clinical science of the brain and the social determinants of the community. By quantifying the invisible obstacles to care, this framework ensures that psychiatric treatment is prioritized as a fundamental human right.

**Disclosure of Interest:**

None Declared

